# No Association between Neck Circumference and Free Triiodothyronine in Euthyroid Men

**DOI:** 10.1155/2021/5570193

**Published:** 2021-04-19

**Authors:** Chaohui Jian, Yiting Xu, Yun Shen, Yufei Wang, Xiaojing Ma, Yuqian Bao

**Affiliations:** Department of Endocrinology and Metabolism, Shanghai Jiao Tong University Affiliated Sixth People's Hospital, Shanghai Clinical Center for Diabetes, Shanghai Key Clinical Center for Metabolic Disease, Shanghai Diabetes Institute, Shanghai Key Laboratory of Diabetes Mellitus, Shanghai 200233, China

## Abstract

**Objective:**

Neck circumference (NC) is a simple anthropometric index for the assessment of upper body obesity. Thyroid hormones are closely related to obesity, body fat distribution indicators, and metabolic parameters. However, there are currently no reports on the association between NC and thyroid hormones in the Chinese population. This study aimed to explore the relationship between NC and thyroid hormones in men with normal thyroid function.

**Methods:**

A total of 737 euthyroid men from Shanghai communities were enrolled. Anthropometric parameters, including NC and waist circumference (WC), were measured. Serum thyroid hormones were measured by electrochemical luminescence immunoassay.

**Results:**

NC, WC, and body mass index (BMI) were significantly positively correlated with serum free triiodothyronine (FT3) (all *P* < 0.05). FT3 levels all presented significant upward trends with the increase in NC, WC, or BMI quartiles (all *P* for trend < 0.05), whereas there were no significant correlations between the three obesity indices and free thyroxine or thyroid-stimulating hormone (both *P* > 0.05). After adjustment for metabolic confounding factors such as age, blood pressure, blood glucose, lipid profiles, and CRP in multiple linear regression analysis, the correlation between FT3 and NC disappeared (standardized *β* = −0.015, *P*=0.705), and FT3 remained significantly associated with WC (standardized *β* = 0.103, *P*=0.012) and BMI (standardized *β* = 0.082, *P*=0.047).

**Conclusions:**

In euthyroid men from Shanghai communities, there was no independent correlation between serum FT3 levels and NC. The trial was registered with ChiCTR1900024011.

## 1. Introduction

Neck circumference (NC), a simple and easy anthropometric index which represents fat deposits in the neck, has a particular advantage in determining upper body obesity compared to body mass index (BMI) and waist circumference (WC) [1]. The explicit anatomic landmark and little interference by breathing and diet make NC measurement more repeatable and less variable. Accumulating studies have demonstrated that NC has the same efficacy as BMI and WC in evaluating obesity-related metabolic abnormalities, such as metabolic syndrome, atherosclerosis, and other cardiovascular diseases [2–4].

Thyroid hormone has been regarded as a regulator of body weight and metabolic status due to its physiological action in regulating energy homeostasis, thermogenesis, oxygen consumption, and glycolipid metabolism [5–8]. Studies have shown that thyroid hormones are significantly correlated with BMI, WC, and other indicators that determine weight status or body fat distribution and metabolic parameters such as lipid profiles, blood pressure, and blood glucose [9–12]. Considering the close anatomical position between NC and the thyroid, NC is often used as a surgical measurement index for indirect assessment of thyroid volume [13–15]. However, in those with normal thyroid morphology, whether NC is associated with thyroid hormones has not been reported in a Chinese population. Compared to women whose fat tends to distribute around the hips and thighs, fat tends to accumulate in the abdomen and neck in men [16]. Therefore, the purpose of this study was to explore the association between NC and thyroid hormones in euthyroid men and assess the impact of thyroid hormones on the clinical use of NC.

## 2. Materials and Methods

### 2.1. Study Population

**Figure 1:**
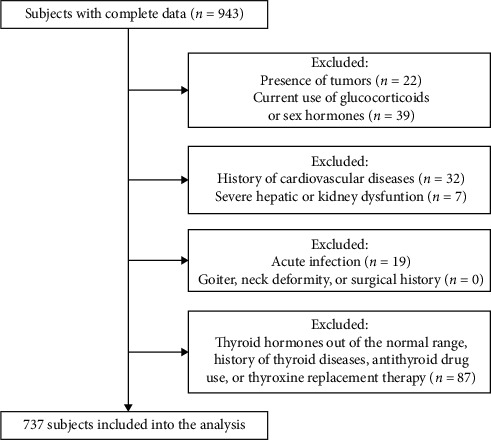
Flowchart of the study population.

A total of 737 euthyroid men were recruited from communities in Shanghai Zhabei from 2015 to 2016. The clinical information was collected through a questionnaire survey by well-trained investigators, which was described in our previous study [2]. Individuals with serum free triiodothyronine (FT3), free thyroxine (FT4), or thyroid-stimulating hormone (TSH) levels out of the normal reference range of the laboratory detection method; history of thyroid diseases, antithyroid drug use, or thyroxine replacement therapy; goiter; neck deformity; surgical history; a validated history of cardiovascular diseases, tumors, or severe hepatic or kidney dysfunction; acute infection; or current use of glucocorticoids or sex hormones were ruled out (Figure 1). This study was approved by the Ethics Committee of Shanghai Jiao Tong University Affiliated Sixth People's Hospital, and informed consent was obtained from all the participants. The trial was registered on the Chinese Clinical Trial Registry (http://www.chictr.org.cn) (registration number: ChiCTR1900024011).

### 2.2. Anthropometric Parameters

All subjects received physical examinations, and measurements of height, weight, NC, WC, and blood pressure were performed using standard operating procedures [2]. BMI was defined as body weight (kg) divided by height (m) squared.

### 2.3. Biochemical Measurements

All subjects underwent a 75 g oral glucose tolerance test or 100 g steamed bread meal test in the morning after a 10 h overnight fast. Fasting and 2 h blood samples were collected. The standard methods used to measure fasting plasma glucose (FPG), 2 h plasma glucose (2hPG), glycated hemoglobin A1c (HbA1c), triglyceride (TG), total cholesterol (TC), low-density lipoprotein cholesterol (LDL-c), high-density lipoprotein cholesterol (HDL-c), C-reactive protein (CRP), and fasting insulin were described previously [2]. The homeostasis model assessment-insulin resistance index (HOMA-IR) was calculated as FPG (mmol/L) × fasting insulin (mU/L)/22.5.

Serum FT3, FT4, and TSH levels were measured by electrochemical luminescence immunoassay (Cobas e601 analyzer, Roche Diagnostics GmbH, Mannheim, Germany). The normal reference ranges of laboratory detection for FT3, FT4, and TSH were 3.1–6.8 pmol/L, 12.0–22.0 pmol/L, and 0.27–4.20 mIU/L, respectively. The intra- and interassay coefficients were <7.0% and <8.0% for FT3, <5.0% and <7.0% for FT4, and <3.0% and <8.0% for TSH.

### 2.4. Statistical Analysis

The statistical analysis was performed using SPSS 20.0 (IBM SPSS Inc., Chicago, IL, USA), and two-tailed *P* < 0.05 was considered statistically significant. Normally distributed and skewed data are shown as the mean ± standard deviation and median (interquartile range), respectively. The comparisons of the normally distributed and skewed data among multiple groups were performed using one-way ANOVA and Kruskal–Wallis H-test, respectively. Spearman analysis was used to explore the correlations between NC and thyroid hormones. The independent associations between NC and thyroid hormones after adjustment for confounding factors were analyzed by multiple linear regression analysis.

## 3. Results

### 3.1. Clinical Characteristics of the Study Participants

Overall, the subjects, aged from 27 to 81 years, had a median age of 61 (56–66) years. The mean levels of BMI, NC, and WC were 24.70 ± 3.12 kg/m^2^, 37.9 ± 2.8 cm, and 88.1 ± 8.7 cm, respectively. The average levels of FT3, FT4, and TSH were 5.15 ± 0.47 pmol/L, 16.83 ± 1.80 pmol/L, and 2.04 (1.51–2.68) mIU/L, respectively. The study participants had a median systolic blood pressure (SBP) of 134 (124–147) mmHg, median diastolic blood pressure (DBP) of 80 (74–87) mmHg, median FPG of 5.84 (5.43–6.52) mmol/L, median 2hPG of 7.77 (6.10–9.91) mmol/L, median HbA1c of 5.7 (5.4–6.1) %, average TC of 5.17 ± 0.91 mmol/L, median TG of 1.52 (1.05–2.27) mmol/L, median HDL-c of 1.23 (1.06–1.43) mmol/L, average LDL-c of 3.16 ± 0.79 mmol/L, and median CRP of 0.84 (0.39–1.62) mg/L.

The individuals were categorized into four groups according to NC quartiles: Q1< 36.1 cm, Q2 36.1–38.0 cm, Q3 38.1–39.9 cm, and Q4 > 39.9 cm. As shown in Table 1, with the increase in NC quartiles, the values of BMI, WC, SBP, DBP, FPG, 2hPG, HbA1c, fasting insulin, HOMA-IR, TG, and CRP showed significant upward trends (all *P* for trend < 0.05), and age and HDL-c decreased significantly (all *P* for trend < 0.05). The levels of TC and LDL-c did not significantly change with increasing NC quartiles (both *P* for trend > 0.05).

### 3.2. The Correlations between NC and Thyroid Hormones

**Figure 2:**
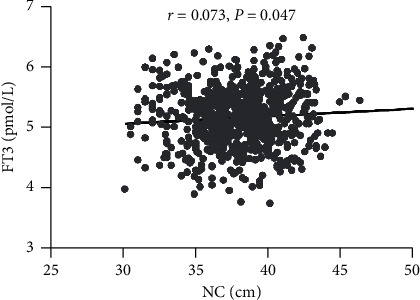
Spearman correlation analysis of neck circumference (NC) and free triiodothyronine (FT3).

Spearman analysis showed that NC was significantly and positively correlated with FT3 (*r* = 0.073, *P*=0.047, Figure 2). There were no significant correlations between NC and FT4 (*r* = −0.005, *P*=0.883) or TSH (*r* = −0.001, *P*=0.988). Furthermore, both WC and BMI were significantly and positively correlated with FT3 (*r* = 0.133 and 0.140, respectively; both *P* < 0.001). Neither WC nor BMI was associated with FT4 (*r* = −0.023 and −0.040, respectively; both *P* > 0.05) or TSH (*r* = −0.020 and 0.004, respectively; both *P* > 0.05).

**Figure 3:**
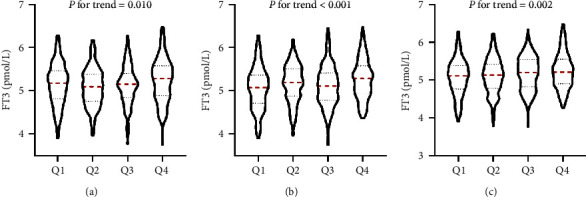
Free triiodothyronine (FT3) levels according to (a) neck circumference (NC) quartiles, (b) waist circumference (WC) quartiles, and (c) body mass index (BMI) quartiles.

As shown in Figure 3, the subjects were divided into four groups according to NC quartiles. The mean levels of FT3 from Q1 to Q4 of NC were 5.15 ± 0.49 pmol/L, 5.08 ± 0.44 pmol/L, 5.14 ± 0.45 pmol/L, and 5.24 ± 0.51 pmol/L, respectively, which presented a significant upward trend (*P* for trend = 0.010). Similarly, when dividing the subjects into four groups based on WC quartiles (Q1 < 82.0 cm, Q2 82.1–88.0 cm, Q3 88.1–94.0 cm, and Q4 > 94.0 cm) or BMI quartiles (Q1 < 22.80 kg/m^2^, Q2 22.81–24.57 kg/m^2^, Q3 24.58–26.50 kg/m^2^, and Q4 > 26.50 kg/m^2^), there were also significant increasing trends in FT3 levels with increasing WC or BMI quartiles (both *P* for trend < 0.05).

### 3.3. Linear Regression Analysis for the Association between NC and FT3

The linear regression analysis in Table 2 showed that FT3 was positively associated with NC, WC, and BMI (standardized *β* = 0.073, 0.164, and 0.169, respectively; all *P* < 0.05) in univariate models. After further adjusting for metabolic confounding factors, including age, blood pressure, HbA1c, HOMA-IR, lipid profiles, and CRP, the positive association between FT3 and NC disappeared (standardized *β* = −0.015, *P*=0.705), and the positive associations between FT3 and WC or BMI remained significant (standardized *β* = 0.103 and 0.082, respectively, and *P*=0.012 and 0.047, respectively).

## 4. Discussion

The current study found that FT3 levels showed significant upward trends with increasing NC, WC, or BMI. After adjustment for metabolic confounding factors, the association between FT3 and NC disappeared, and the independent and positive associations between FT3 and WC or BMI remained significant.

Thyroid hormones maintain body homeostasis through the regulation roles on the basal metabolic rate, heat production, oxygen consumption, and lipolysis. Consequently, hyperthyroidism and hypothyroidism are often accompanied by the fluctuations in basal metabolic rate and weight and even lead to glucose and lipid metabolism disturbance and cardiovascular diseases [17]. Previous studies have demonstrated the associations of obesity and body fat distribution-related parameters with thyroid hormones. Roef et al. conducted a cross-sectional study in 2315 euthyroid healthy subjects and found that both FT3 and FT3/FT4 were significantly and positively correlated with BMI, WC, and metabolic syndrome components and that there were significant and inverse associations between FT4 and BMI, WC, and TG. Moreover, they also observed significant and positive associations between FT3/FT4 and the fat-related inflammatory factors interleukin 6 and CRP [18]. A study in Korea, which enrolled 36,655 subjects with normal thyroid function and ultrasound data, showed that, in multiple linear regression analysis of men, FT3 was positively related to WC, but negatively related to the skeletal muscle mass index. However, neither FT4 nor TSH was significantly correlated with WC [9]. Another study in 941 euthyroid healthy men assessed body composition and muscle cross-sectional area by dual-energy X-ray absorptiometry and peripheral quantitative computed tomography and observed that BMI, fat mass, and leptin levels increased in higher FT3 quartiles [19]. Given the complex regulatory mechanism of thyroid hormones and the difference in the study populations, studies regarding the relationships between thyroid hormones and obesity or fat distribution-related indicators did not reach a consistent conclusion. In our study, WC and BMI were significantly and positively related to serum FT3 levels, irrespective of metabolic confounding factors, which was consistent with previous reports.

Evidence indicated that NC was not merely a simple indicator for the upper body fat accumulation and was also found to be associated with diabetic complications and effective in identifying subclinical atherosclerosis and nonalcoholic fatty liver disease screening [20–22]. A few studies have compared the NC values of those with subclinical hypothyroidism and euthyroid individuals [23–26]. Belen et al. observed that NC in subclinical hypothyroidism patients was significantly higher than that in gender-matched healthy subjects [23]. Another study in overweight/obese patients found no significant difference in NC levels between subclinical hypothyroidism patients and euthyroid subjects [24]. In the study conducted by Mousa et al., 99 Hashimoto's thyroiditis patients with thyroid hormones within the normal ranges and 202 control subjects were matched according to age, sex, BMI, smoking, thyroid nodules, and menopausal status. The results showed that the NC levels in Hashimoto's thyroiditis group were not significantly different from those in the control group [25]. A cross-sectional study with 11,224 euthyroid subjects in Brazil presented that NC was not related to TSH in men, even before the adjustment for age, sex, race, education, thyroid peroxidase antibody, diabetes, cardiovascular disease, glomerular filtration rate, and lifestyle [26]. The present study was the first to demonstrate the relationship between NC and thyroid hormones in Chinese euthyroid men. Unlike the study in Brazil, which only tested TSH, the current study detected FT3, FT4, and TSH as three thyroid hormones in euthyroid men and found that NC, WC, and BMI were all significantly and positively related to FT3. However, after adjustment for confounding factors, the association between NC and FT3 disappeared, and the independent association between WC or BMI and FT3 remained significant. Since WC and BMI are simple indicators reflecting visceral and systemic fat content, respectively, the space for fat deposition in the neck is much smaller than that in the abdomen and the whole body. The differences between abdominal and systemic fat content and neck fat content may explain the disappearance of the correlation between NC and FT3 after adjustment for metabolic factors. Furthermore, the study participants were all euthyroid individuals, and small fluctuations in serum thyroid hormones may not cause significant changes in neck fat and NC. Therefore, only WC and BMI remained significantly and positively correlated with serum FT3 levels after adjustment for metabolic confounding factors.

Due to the difference in the study populations, there are no consistent conclusions on the relationships between thyroid hormones and NC. Previous studies investigated the related mechanisms of TSH in adipocyte and adipose tissue metabolism [27–29], but the underlying mechanism of the relationship between FT3 and NC has been rarely reported. Elevated NC indicates a relative accumulation of neck fat, and paradoxically, high FT3 levels increase energy consumption at rest and prevent further fat accumulation. Thus, whether the increase in FT3 is due to the regulation of FT4 and TSH or the direct dialogue between FT3 and local adipose tissue needs more in vitro studies to verify.

Our study was limited in the single-center cross-sectional design. The results need to be verified in a multicenter, large-sample population. Moreover, the exclusion of thyroid diseases and goiter was only based on the medical history and physical examination, and the lack of direct thyroid morphology by ultrasound might affect the results. In addition, whether there is direct crosstalk between FT3 and neck adipose tissue needs to be revealed by further basic research.

## 5. Conclusion

In euthyroid men from communities in Shanghai, there was no independent association between serum FT3 and NC.

## Figures and Tables

**Table 1:** Clinical characteristics of the study participants.

Variables	NC quartiles				*P* for trend
	Q1	Q2	Q3	Q4	
*N*	184	191	179	183	—
Age (years)	62 (55–67)	62 (57–66)	61 (57–65)	59 (53–65)	0.026
BMI (kg/m^2^)	22.61 ± 2.68	23.38 ± 2.05	25.14 ± 1.97	27.76 ± 2.84	<0.001
NC (cm)	34.2 ± 1.4	37.1 ± 0.6	39.0 ± 0.5	41.4 ± 1.2	<0.001
WC (cm)	81.4 ± 7.7	84.8 ± 5.9	89.9 ± 5.7	96.3 ± 7.3	<0.001
SBP (mmHg)	132 (119–142)	131 (121–145)	133 (125–146)	140 (129–151)	<0.001
DBP (mmHg)	79 (72–85)	79 (72–86)	81 (75–88)	84 (77–91)	<0.001
FPG (mmol/L)	5.70 (5.36–6.30)	5.87 (5.44–6.49)	5.84 (5.47–6.45)	6.03 (5.56–6.89)	0.003
2hPG (mmol/L)	7.30 (5.66–8.86)	7.72 (6.23–9.72)	7.69 (6.21–9.76)	8.49 (6.34–11.88)	0.003
HbA1c (%)	5.6 (5.4–6.0)	5.7 (5.4–6.0)	5.7 (5.4–6.0)	5.8 (5.5–6.3)	0.006
Fasting insulin (mU/L)	6.66 (4.50–9.73)	7.42 (5.20–9.89)	9.02 (6.75–13.61)	11.64 (8.15–15.81)	<0.001
HOMA-IR	1.76 (1.18–2.65)	1.98 (1.41–2.84)	2.45 (1.81–3.69)	3.40 (2.17–4.90)	<0.001
TC (mmol/L)	5.06 ± 0.79	5.28 ± 1.00	5.16 ± 0.92	5.16 ± 0.92	0.139
TG (mmol/L)	1.25 (0.90–1.98)	1.47 (1.04–2.22)	1.61 (1.10–2.47)	1.78 (1.26–2.52)	<0.001
HDL-c (mmol/L)	1.34 (1.11–1.60)	1.25 (1.12–1.50)	1.21 (1.03–1.38)	1.15 (0.98–1.31)	<0.001
LDL-c (mmol/L)	3.06 ± 0.71	3.20 ± 0.89	3.21 ± 0.77	3.18 ± 0.75	0.243
CRP (mg/L)	0.58 (0.32–1.26)	0.79 (0.38–1.61)	0.97 (0.39–1.91)	1.03 (0.50–1.88)	<0.001

Data were expressed as mean ± standard deviation for normally distributed variables or the median (interquartile range) for skewed distribution variables. NC: neck circumference; BMI: body mass index; WC: waist circumference; SBP: systolic blood pressure; DBP: diastolic blood pressure; FPG: fasting plasma glucose; 2hPG: 2 h plasma glucose; HbA1c: glycated hemoglobin A1c; HOMA-IR: homeostasis model assessment-insulin resistance index; TC: total cholesterol; TG: triglyceride; HDL-c: high-density lipoprotein cholesterol; LDL-c: low-density lipoprotein cholesterol; CRP: C-reactive protein.

**Table 2:** Linear regression analysis for the associations of FT3 with NC, WC, and BMI.

Variables	NC		WC		BMI	
	Standardized *β* (*t*)	*P* value	Standardized *β* (*t*)	*P* value	Standardized *β* (*t*)	*P* value
Unadjusted model	0.073 (1.989)	0.047	0.164 (4.500)	<0.001	0.169 (4.635)	<0.001
Multivariate model	–0.015 (–0.378)	0.705	0.103 (2.520)	0.012	0.082 (1.990)	0.047

Multivariate model: adjustment was for age, SBP, DBP, HbA1c, HOMA-IR, TC, TG, HDL-c, LDL-c, and CRP. FT3: free triiodothyronine; NC: neck circumference; WC: waist circumference; BMI: body mass index; SBP: systolic blood pressure; DBP: diastolic blood pressure; HbA1c: glycated hemoglobin A1c; HOMA-IR: homeostasis model assessment-insulin resistance index; TC: total cholesterol; TG: triglyceride; HDL-c: high-density lipoprotein cholesterol; LDL-c: low-density lipoprotein cholesterol; CRP: C-reactive protein.

## Data Availability

The datasets generated for this study will not be made publicly available because the ethical approval obtained for this study prevents the human data being shared publicly to protect patients' privacy. Requests to access the datasets should be directed to Yuqian Bao (yqbao@sjtu.edu.cn). This would be passed to the ethics committee who will decide whether they can access the data directly.
